# Interventions that prevent or reduce obesity in children from birth to five years of age: A systematic review

**DOI:** 10.1177/1367493520917863

**Published:** 2020-04-15

**Authors:** Katia Narzisi, Joan Simons

**Affiliations:** 1 Faculty of Wellbeing, Education and Language Studies, The Open University, Walton Hall, Milton Keynes, UK

**Keywords:** Childhood obesity, effective, interventions, overweight, systematic review, under-fives

## Abstract

Childhood obesity worldwide affects 5.6% or 38.3 million children under five years of age. The longer children are overweight or obese, the more likely they are to become obese adults with all the contingent morbidity involved. An extensive number of preventive interventions to combat childhood obesity have been carried out worldwide. This article reports a systematic review of interventions aimed to reducing or preventing obesity under-fives. The search was performed with six different databases: Web of Science, PsycINFO, Cochrane, PubMed, Medline, and CINAHL. Studies meeting the inclusion criteria were independently assessed using Joanna Briggs Institute methodology. Thirty studies involving 23,185 children across nine countries were included. Twenty-two were randomised controlled trials, and 8 quasi-experimental pretest/post-test design with comparison. These studies fell into four different categories: home-based interventions with family involvement (*n* = 12), preschool/early childhood settings (*n* = 9), multicomponent interventions across multiple settings (*n* = 6) and healthcare setting (*n* = 3). Future research should focus on increasing the accessibility of education on diet and physical activity for deprived families as well as the cultural acceptability of interventions to prevent childhood obesity.

## Background

Early childhood overweight and obesity is a major health concern affecting nearly a quarter of children in the United States, with similar rates in Europe, Canada and Australia (Morris et al., 2015), with Shakleton et al. (2018) reporting that New Zealand has among the highest rates in the world. Rudolf et al. (2019) has reported that as many as 10% of children in the United Kingdom are obese starting school. If obesity rates are resistant in school-aged children, the focus on intervention for preschool children is likely to have more impact due to the potential to curb obesity rates earlier by intervening in younger children’s lives.

In response to these rates of childhood obesity, the rate of published studies has grown incrementally in a search for an answer as to how to prevent obesity. The growth of disparate studies in various countries has created the information necessary for systematic reviews of the field, to achieve clarity on what works. Wang et al. (2015) carried out a systematic review looking for what childhood obesity prevention programmes efficacy and concluded that school-based interventions can help prevent obesity in children. They report that school is seen as a prime site for childhood obesity interventions; however, Gutin (2008) suggest that interventions that have been found to be effective at first lose their effect during the summer. Hung et al. (2015), who carried out a meta-analysis of school-based obesity prevention programmes, found limited efficacy of decreasing childhood obesity.

In response to these challenges, there has been a significant growth in studies of interventions to prevent obesity in the under-fives since 2010, focusing on different areas such as infant feeding, suggesting there is a critical window for adiposity development in the first three months of life (Breij et al., 2017); breastfeeding has been found to be more effective in reducing obesity risk in children than interventions promoting healthy eating or physical activity. Household chaos has been found to be associated with greater infant weight gain at 12 months (Khatiwada et al., 2018). A Scottish study (Macintyre et al., 2018) found some evidence of association with early exposure to artificially sweetened beverages (aged 4–5 years) and risk of obesity (aged 7–8 years). Skouteris et al. (2011) suggested that there is still a need for more studies evaluating targeted obesity prevention studies in preschool children.

### Reviews on prevention and management of weight in children under five years

In the search for higher quality evidence, there has also been a growth in systematic reviews of obesity-related interventions in the under-fives in an attempt to identify optimal intervention design. A range of reviews (Bleich et al., 2018; Bluford et al., 2007; Bond et al., 2011; Brown et al., 2019; Campbell and Hesketh, 2007) show that considerable efforts to tackle childhood obesity have been made. However, there are a number of shortcomings in relation to identifying effective interventions in the under-fives: Bleich et al. (2018) looked at interventions from birth up to 18 years of age and therefore did not make recommendations for different age groups. Bond et al. (2011) focused only on weight management schemes. Bluford et al. (2007) carried out a comprehensive review but focused on 2- to 6-year-olds, which therefore included children who are already in school. Blake-Lamb et al. (2016) looked at the period of conception to 24 months, and Ling et al. (2016) interpreted ‘preschool’ to mean an older age group, which included studies with children of 7 years of age. Campbell and Hesketh (2007) included studies up to 2006 and called for substantially more work in this area. This study adds a comprehensive review of recent interventions to prevent or reduce childhood obesity focusing on children up to five years of age.

### Study objective

This systematic review answers these questions: What interventions are being carried out to promote healthy weight in children under the age of five and how are effective outcomes achieved? It aims to add to existing literature by presenting a systematic review of interventions for under-fives aimed at preventing or treating childhood obesity.

### Inclusion and exclusion criteria

The inclusion and exclusion criteria guided the PICO(S) elements described above, as well as the study objectives. The articles that reported an intervention with (a) the main target population being children from birth up to the age of five years; (b) were designed with randomised controlled trials, cluster randomised trial, single-blind trial, double-blind trial, pre- and post-test, community-based intervention with pre- and post-test measures; (c) had both baseline and post-intervention measures; (d) included at least one anthropometric outcome measure, body mass index (BMI), BMI percentile and *z*-score (standard deviation), waist circumference, skinfold and body fat and (e) evaluated a lifestyle intervention, which aimed at changing health through targeting two or more behaviours, such as diet, physical activity, sedentary behaviour, sleep patterns and screen time (as defined by Martin et al., 2012, and World Health Organisation (WHO), 1998).

Studies were not included if (a) they only presented lifestyle outcomes, without anthropometric measures, (b) they targeted one or more diseases linked with obesity, (c) they mainly focused on children over five years of age, (d) they were not published in English and (e) only the abstract was available to the researchers. Pilot studies that reported effective outcomes were included as they helped to examine the feasibility of a specific approach that is aimed at a larger population. They provided useful insight on processes of recruitment, retention, analysis of data and new methods or interventions (Leon et al., 2011).

## Method

This review used the Joanna Briggs Institute (JBI) methodology (Schultz and Florence, 2007), which included the JBI data extraction tool and a range of JBI quality appraisal tools which focused on the feasibility, appropriateness, meaningfulness and effectiveness of healthcare interventions. For the purpose of this article, following the WHO growth standards recommendations (WHO, 2006) for children from birth to the age of five years, ‘overweight’ was defined as BMI between 85th and 94th percentile and ‘obesity’ as BMI over 95th percentile for age and sex.

### Search strategy

A comprehensive search strategy was employed drawing on the following stages: identify existing reviews focused on childhood obesity interventions and systematic searches of six databases from their inception to June 2018: Web of Science (from 1900), PsycInfo (from 1975), Cochrane (from 1965), PubMed (from 1966), MedLine (1946) and CINAHL (from 1982). The search was defined through PICO(S), based on these four components: (i) P(population) included terms restricted to infants and children; (ii) I(intervention) and C(comparison) with terms related to the intervention, such as promotion, control, reduction, health promotion, lifestyle or physical activity; (iii) O(outcome) included terms like obesity reduction, overweight, weight maintenance and (iv) S(studies) with terms related to the type of designs, such as RCT. With these four major components identified, the terms were converted to MESH terms and subject descriptors. The search strategy was consistently applied to each database, according to their interfaces.

### Study selection

The selection of the studies was approached in three stages. Titles were initially screened and reduced through titles and abstracts and then through titles and abstracts both researchers independently examined the possible inclusion. Full articles were obtained and screened by the same researchers for inclusion in the review, and disagreements were resolved through discussion.

### Data extraction and synthesis

Data were extracted by two independent reviewers. The data extraction form that has been developed following the standardised data extraction tool from JBI-MAStARI (Schultz et al., 2007) was used to extract the data in a consistent way for all studies reviewed. The data extracted includes specific details about the interventions, populations, study methods and outcomes of significance to the review question and specific objectives.

### Quality appraisal

Two independent reviewers assessed the quality of each study, testing its validity before being included in the review. To assess the validity of the articles, the JBI critical appraisal tools developed by JBI were used (Schultz et al., 2007). Each study’s methodological quality and the extent to which each study addressed the possibility of bias in its design, conduct and analysis was assessed. One of three different JBI checklists, for Randomised Controlled Trials, Quasi-Experimental Studies (non-randomized experimental studies) and Cohort Studies were used. The outcomes of the appraisal were used to inform the interpretation of the results of the studies. Please see supplementary information for the outcomes of the critical appraisal process.

## Results

**Figure 1.**
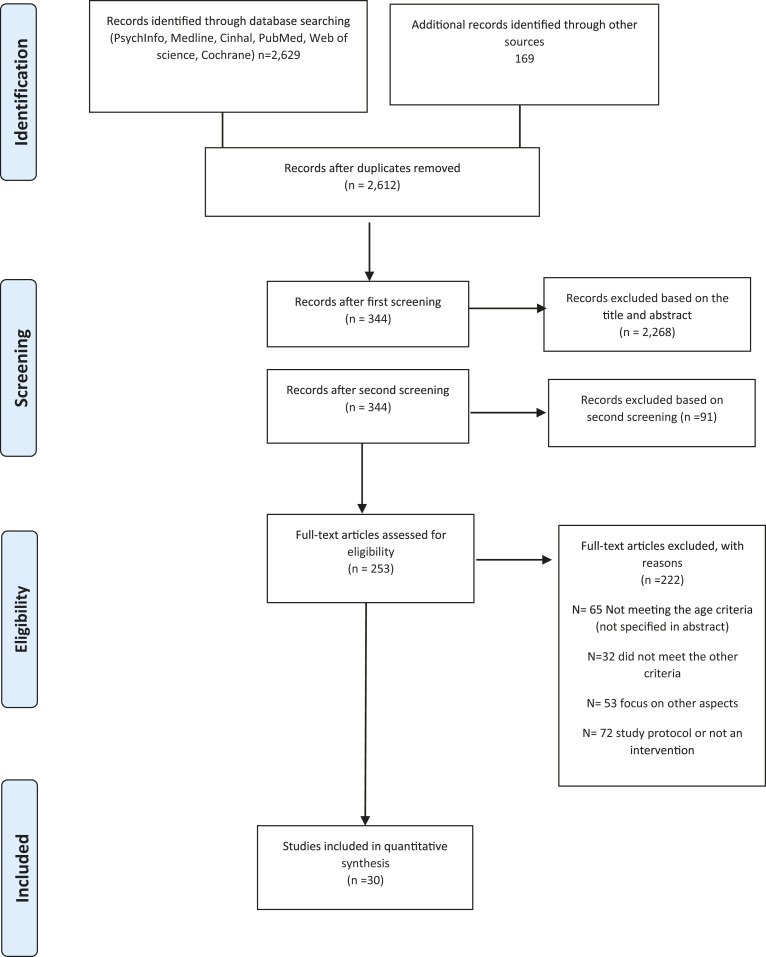
PRISMA 2009 flow diagram.

The study selection process with results are presented in the Preferred Reporting Items for Systematic Reviews and Meta-Analyses (PRISMA) diagram in Figure 1. Through the six databases used, 2629 articles were identified. These were reduced to 344, through their titles and reading of the abstracts. The abstracts were then screened by one researcher, through the second reading of the abstracts, resulting in 91 articles being excluded as they did not meet the inclusion criteria. A total of 253 articles were selected for analysis by two independent researchers. The articles that were excluded by one researcher but included by the other researcher were discussed and an agreement was found. Both researchers agreed on the inclusion of 30 studies.

Of the 30 selected studies, there were 25 unique interventions. The number of children involved in the studies was 23,185 and the duration of the interventions ranged from 14 weeks to four years, with follow-up from none to four years in length. The majority of the studies were undertaken in the United States (17) and Australia (6), with 1 each in the United Kingdom, Belgium, The Netherlands, Sweden, Canada, New Zealand and China. There were 22 RCTs and 8 quasi-experimental studies. Of the 30 studies, there were 7 pilot studies, the majority of which (5) were RCTs (further details in Supplementary Information 1).

### Home-based interventions with family involvement

Six home-based studies used interventions focused on promoting healthy feeding practices and parenting skills with varying degrees of success. Healthy beginnings (*n* = 667) provided eight home visits from trained nurses around growth milestones resulting in a significantly lower mean BMI in the intervention group than in the control group (Wen et al., 2012). Paul et al. (2011) conducted a pilot study, including 160 mother and child dyads with a 2 × 2 design, and the group that received both interventions had lower weight for length percentiles (*p* = .009).

An early childhood trial called NOURISH was the focus of an RCT by Daniels et al. (2012), which applied cognitive behavioural theory to underpin a comprehensive skills-based programme on early feeding and parenting practices. The intervention involved children aged four to six months (*n* = 698), who attended 2 three-month education modules led by a dietitian. At 13 months, children in the intervention group had lower BMI *z*-scores compared to the control group, but a later study by Daniels et al. (2013) reported it was not sustained at age two years.

Campbell et al. (2013) carried out an RCT with infants and their mothers (*n* = 542) to assess the effectiveness of a parent-focused intervention on infant’s obesity risk behaviours and BMI. The intervention involved six 2-hour dietician delivered sessions over 15 months on parental knowledge, skills and social support around infant feeding, diet, physical activity and television (TV) viewing. At mean age 19.8 months, intervention group children consumed fewer grams of sweet snacks and viewed fewer daily minutes of TV. Limitations of the study were over-representation of highly educated mothers and the possibility of a desire to provide desirable responses when recalling diet and TV viewing.

The only study on bottle weaning by Bonuck et al. (2014) aimed to prevent childhood obesity using ‘Women, Infant, Children’ (WIC) nutritionists who delivered an educational intervention which included messages about healthy weight, dental caries and iron deficiency anaemia effects from bottle feeding. The intervention group had reduced daily energy intake from milk bottles and reduced total energy intake compared to controls. Despite the reduced energy intake in the intervention group, there were no effects on risk of overweight status. One limitation was that participants were not masked to treatment status, so social desirability status may have affected reported bottle use.

Six studies focused on physical activity and exercise in children aged two to five years with a home-based component. The only study aiming to reduce obesity, ‘Learning About Activity and Understanding Nutrition for Child Health’ (LAUNCH) by Stark et al. (2014), was a six months long pilot RCT, which was home and clinic based. It was a behavioural intervention with children aged two to five years (*n* = 18). These sessions were run in clinic for both parents and children and then applied in the home environment with the supervision of research team members. LAUNCH showed a decrease in BMI at month 6 (post-treatment), which was maintained after 12 months.

The remaining five studies aimed to prevent obesity. Ostbye et al. (2012) carried out an eight-month RCT study called KAN-DO (*n* = 400) with children aged two to five years, involving instruction on parenting style and skills alongside techniques for stress management and education about healthy behaviours. Although changes noted were not statistically significant, there were reductions in instrumental feeding and TV snacks and improvements in emotional feeding, fruit and vegetable intake and number of dinners eaten in front of the TV.

Harvey-Berino and Rourke’s (2003) study included preschool Native American children (*n* = 43). In both groups, a peer educator delivered the programme, which was adapted to make it culturally appropriate. Mothers were given a US$25 gift certificate for a local mall when all measures were complete. At week 16, changes in the weight for height *z*-score indicated that children in the obesity prevention group gained less weight than those in the parenting support intervention. Woo Baidal et al. (2017) carried out a two-year obesity prevention quasi-experimental study with low-income preschool children (*n* = 1461), aged two to four years, as part of the Massachusetts Childhood Obesity Research Demonstration initiative. BMI scores were extracted from WIC records and two cross-sectional samples of parents were interviewed on behavioural outcome measures. There were small but not statistically significant changes to BMI *z*-score in both intervention sites. There were no changes in fruit and vegetable intake or screen time.

A 10-week RCT with preschool children (*n* = 201) in New Zealand had mixed results (Skouteris et al., 2011). Weekly 90-minute workshops related to nutrition, physical activity and behaviours, including guided healthy play and snack time, resulted in significant positive group effects for vegetables and snack food intake as well as satiety responsiveness. There was no effect on physical activity, sedentary behaviour or BMI z-score.

The only study that had a participatory research approach was a one-year pilot intervention by Davison et al. (2013) with children aged two to five years (*n* = 453). It was a multisite study, involving low-income Head Start pupils and the community, through campaigns and families involving a health communication campaign, revised BMI letters, family nutrition counselling, parents connect for healthy living programme and a child programme. Children presented marginally lower BMI *z*-scores and significantly lower rates of obesity with greater time of moderate activity and lower rates of sedentary activity. Post-intervention parents reported greater self-efficacy to provide healthy foods.

### Preschool/early childhood setting

This group included nine studies with six interventions and three pilot studies. Herman et al. (2012) carried out a pilot study to test the ‘Eat Healthy, Stay Active’ programme, using pre- and post-test intervention (*n* = 112 preschool children). The goal of the intervention was to increase knowledge and awareness on health behaviours, and it was done through low health literacy training programme for obesity prevention. There was a significant reduction in BMI in children and in the proportion of obese children and adults. The weakness of this intervention is that no control group was used to verify the outcomes.

Jones et al. (2011) developed a two-arm parallel pilot cluster RCT ‘Jump Start’ in which they tested the efficacy of a movement skill development programme (*n* = 97) with two parts, one for the development of staff and structured and unstructured sessions for children, implemented three times a week for 20 minutes each time. A statistically significant difference between the intervention and control group was reported for the sum of the five movement skills assessed.

Hip-Hop to Health Jr. was a 14 weeks school-based pilot cluster RCT aimed to prevent obesity in three to five years old Latino children and their parents, with family-based components. The study has been reported post-intervention and at one (*n* = 618) and two years follow-up (*n* = 402) (Fitzgibbon et al., 2005, 2011; Kong et al., 2016). The intervention was only partially successful. The importance of working with parents was an aspect frequently highlighted, but their willingness to be actively involved in this research project was still challenging. Nonetheless, after the intervention, the BMI decreased in the intervention group and it changed by .09 in the control group. However, after one year, the BMI had increased in both the intervention and the control group, but by year two follow-up, the intervention children had significantly smaller increases in BMI compared to the control children, suggesting that the benefit of such an intervention may take some time to be realised.

Winter and Sass (2011) carried out the only study with a school readiness approach, called Healthy and Ready to Learn (*n* = 405), which lasted 24 weeks, and included parents and teachers. Five specific behaviours that influence children’s growth were the focus of the intervention. There were statistically significant differences in children’s physical activity levels, height, gross motor skills and receptive language development, but there was no statistically significant difference in children’s BMI.

Bellows et al. (2013) carried out an RCT in four American Head Start Centres with children aged three to five years (*n* = 263). The physical activity intervention was classroom sessions four days a week for 15–20 minutes. Children also wore pedometers on six separate days and parents were paid US$20 for completing the pedometer log. There were statistically significant changes to the gross motor skills of children in the intervention group. However, no intervention effect was found for physical activity levels or weight.

Another quasi-experimental obesity prevention study called Miranos! was carried out by Yin et al. (2012) with Mexican American children (*n* = 423) in a deprived area of Texas. A system approach aimed to modify daily eating and physical activity behaviours and to provide an interactive, supportive learning environment for children. Seven parents were trained and paid a stipend to train to be peer educators to deliver the home intervention. The combined home and centre-based intervention group had statistically significant less weight gain than comparison children. There was an increase in gross motor skills in the intervention groups, which was not statistically significant.

### Multicomponent interventions across multiple locations

The largest study was the Romp & Chomp (De Silva-Sanigorski et al., 2010), a community-wide intervention aimed at 12,000 children from birth to five years. Activities focused on building changes within the community and influencing policy. It is a repeat cross-sectional quasi-experimental design with pre- and post-measures. There was a reduction in zBMI; in 2-year-olds, the zBMI went from .71 to .68, and in the 3.5-year-olds, it went from .67 to .54, with a significantly lower intake of packaged snacks and fruit juices.

Zhou et al. (2014) report on a 12-month long quasi-experimental pre- and post-design, testing the effectiveness of a multi-setting intervention that included childcare centres, families and community to promote healthy behaviours and physical fitness in preschool Chinese children (*n* = 357) aged three to five years. In the childcare centres, the physical activity was increased as well as the quality of food services. They presented lowered body fat percent, fat mass and body weight and increased muscle mass. However, BMI scores and weight did increase in the post-test measures, for both the intervention group and the control group.

Heathy Inside-Healthy Outside was delivered through a 6-month RCT in subsidised childcare centres, to children (*n* = 307) aged two to five years, their teachers and the families (Natale et al., 2014). Intervention centres received healthy menu changes and family-based education on increased activity and fresh produce. There were significant changes in BMI *z*-scores, from .67 to .57, with 97% of the children who had a normal weight at baseline still had a normal weight after 12 months and 4% of the children who started the programme overweight had a normal weight after 12 months. However, at follow-up, BMIz had gone up to .45.

Verbestel et al. (2013) tested a 12-month pilot cluster RCT on toddler’s BMI *z*-scores and reported activity and diet. It was a family-based intervention implemented through day-care centres. The intervention had a positive effect on BMI *z*-score, which decreased in both groups, but decreased more in the intervention group compared with the control group.

Pettman et al. (2014) conducted a quasi-experimental study to explore the ability of the Eat Well Be Active programme, with children aged four to five years (*n* = 1626 with 1198 at follow-up). Results showed that between 2006 and 2009, mean zBMI was significantly lower in both the intervention group and the comparison group; however, there was no statistically significant difference between the intervention group and the comparison group. The use of cross-sectional measurements rather than individual measurements in Pettman et al.’s study suggests that changes could not be solely attributable to their intervention.

Reilly et al. (2006) carried out a cluster RCT that focused on a physical ability intervention to prevent obesity in young children (*n* = 545), in 36 nurseries in a deprived area of Scotland in their preschool year. The intervention involved three 30-minute sessions a week on physical activity over 24 weeks and home-based education aimed at increasing physical activity through play and reducing sedentary behaviour. No significant effect was found of the intervention on physical activity, sedentary behaviour or BMI. The authors suggest the intervention was inadequate in relation to physical activity, and more extensive work with parents might have been more successful.

### Healthcare setting

Both Bocca et al. (2012) and Quattrin et al. (2012) recruited either overweight or obese children in studies to treat obesity. In Bocca et al. (2012), the objective was to assess the effects of a multidisciplinary intervention programme for 75 children aged three to five years compared with a usual-care programme. In Quattrin et al. (2012), the objective was to test a family-based intervention with 96 children, administered through 10 sessions of 60 minutes over six months in the clinics, with a physical activity goal of 60 minutes per day for children along with individual sessions with the coach about healthy eating and physical activity. Both interventions had effective outcomes; in Bocca et al. (2012), significant decrease in BMI was recorded but not in physical activity. In Quattrin et al. (2012), children in the intervention group had greater % over Body Mass Index and zBMI decreases at three and six months compared to those assigned to the control group.

Döring et al. (2016) carried out a population-based RCT over 39 months, in child healthcare centres in Sweden. First-time parents receiving preventive services were recruited by trial nurses when their babies were five to six months of age. The intervention involved one group session and eight individual sessions with a nurse trained in motivational interviewing, focusing on healthy food habits and physical activity. There were no statistically significant differences in children’s BMI, waist circumference or prevalence of overweight.

## Discussion

Public Health England (PHE, 2018) advocates for a community approach to tackling childhood obesity, underpinned by the stance that obesity is not an individual problem but a societal problem. These findings are supported by studies in this review that reported on community approaches because they engage layers of society surrounding children such as children’s services, schools and homes and thereby support behavioural change (Campbell and Hesketh 2007; de Silva-Sanigorska et al., 2010; Reilly et al., 2006).

A number of studies reported marginal effects that were not statistically significant (Ostbye et al., 2012; Woo Baidal et al., 2017); however, most studies did not have long-term follow-up of the interventions. Also, the nature of the age group, under-fives, means that dietary habits formed at this age are likely to lifelong influence in relation to food preferences, making interventions at this stage of life very cost-effective (Brown et al., 2019).

There appears to be a challenge, however, in the recruitment of deprived families, with some studies reporting that the desired or planned focused study group, that is, deprived children and families, were not the sample they recruited (Ostbye et al., 2012; Skouteris et al., 2011), suggesting limited usefulness of the findings. Despite this, there is a growing focus in studies on childhood obesity to focus on areas of deprivation. Many American studies in this review, for example, focused specifically on children attending Head Start centres from areas of deprivation. In the United Kingdom, it has been found that the rate of childhood obesity in areas of deprivation is double the rate in areas of affluence (PHE, 2018).

It was noted in some studies (Campbell et al., 2013; Fitzgibbon et al., 2011) that when barriers to access for parents were not addressed, the dropout rate was negatively affected or the study had missing data. In a systematic review by Ling et al. (2016), it was suggested that interventions should focus on parents as change agents. Therefore, there is a need to address accessibility or motivational issues to enable parents to participate. This was overcome by Bellows et al. (2013) and Harvey-Berino and Rourke (2003) who provided small monetary incentives to parents. There is a need when planning studies to prevent or treat childhood obesity to remove any recognisable barriers to access for low-income families invited to participate in the study. Another angle was taken by Davison et al. (2013) who employed a participatory research approach developed with and by parents. As a result, the intervention with low-income families was effective.

Cultural sensitivity was also found to be an influencing factor in studies in this review (Harvey – Berino and Rourke’s, 2003) and Yin et al.’s (2012) study with Native American children and Mexican American children. The findings of two recent RCTs with Hispanic and Latino families (Crespo et al., 2018; Hull et al., 2016) suggest the need for tailoring childhood obesity interventions to special populations to optimise engagement.

A number of obesity prevention interventions in this review (*n* = 5) focused on new mothers or mothers and their infants, with each study managing to have a positive impact on nutrition. Daniels et al. (2012) found that anticipatory feeding guidance and responsive feeding timed before babies were weaned could be an effective time to intervene in feeding practices that prevented babies becoming overweight or obese. Paul et al (2011) found that two interventions which taught new mothers to discriminate between hunger and other sources of distress, as well as hunger and satiety cues, resulted in lower weight for length at 1 year. A systematic review by Blake-Lamb et al. (2016) showed that new mothers are receptive to health-related messages and suggest that obesity interventions begun early in life may have the greatest preventive effect; however, interventions during infancy need to be followed up to determine long-term benefits.

Multilevel studies (de Silva-Sanigorski et al., 2010; Natale et al., 2014; Zhou et al., 2014) were more likely to be effective because they included different elements of society that surround children, such as schools, and those who are highly influential in the early development of children, such as their parents and childcare staff (Winter and Sass 2011).

Finally, the need for effective studies for preschool children is reinforced by a study by Turner et al. (2016) who explored the views of school health professionals and their capacity to deal with childhood obesity. Findings were that although they have an important role to play in managing childhood obesity, efforts to address child weight were limited by a lack of capacity, lack of clear protocols, challenges of engaging parents and insufficient training in childhood obesity and related lifestyle issues. These finding reinforce the necessity to invest in interventions that prevent obesity in preschool children.

## Limitations

The nature of this systematic review only captures studies with children up to the age of five years as the term ‘preschool’ was imprecise because the age at which children start school varies from country to country. This meant that other potentially relevant studies of preschool children may have been missed. Another limitation is the small number of well-designed full RCTs. The selection of the databases may also have limited the range of articles included in the review, and the articles presented here are published only in English, which excludes publications in any other language. This may limit the range of countries and interventions included in this review. Different search terms could have increased the number of infant feeding studies (such as work on responsive feeding), from five, and could have highlighted more strongly the potential for obesity prevention interventions to be undertaken during infancy.

## Recommendations for future research

The most efficient level of intensity in relations of how many components a study needs to make an impact needs to be further investigated alongside the length of the intervention and most appropriate time needed for follow-up. Another aspect that should be addressed through research is the rigour in reporting data to enable comparisons to be made across studies. Studies were excluded from this review because not all information around the methodological standards followed were reported appropriately.

In the selected studies, only one was based in the United Kingdom (Reilly et al., 2006) despite PHE (2018) reporting that the proportion of preschool children with weight issues needs to be tackled. The rate of childhood obesity in deprived areas of England is double the rate of affluent areas (PHE, 2018). There is a clear need to design accessible, multicomponent studies to include low-income families with preschool children, in interventions to prevent obesity.

## Implications for practice

The aim of this review was to examine the existing interventions and their outcomes to identify guidance for practitioners and policymakers on how to promote healthy weight in children under five years of age. There is a growing body of evidence that indicates the preschool years as an important window of opportunity to prevent children of school age entering the education system either overweight or obese. Resources need to be put in place to ensure that vulnerable families benefit from this evidence by being able to access support with parenting skills that help influence children’s diet and physical activity levels to enable them to have childhoods that are unimpaired by childhood obesity.

## Conclusion

The only constant in a child’s life is their parents and carers; therefore, engaging parents in attempts to prevent childhood obesity seems an obvious move. A particularly influential time period appears to be new motherhood when mothers are receptive to health messages, as exemplified by the studies focusing on infant feeding and weaning practices. The ability to influence obesity risk factors at this early age suggests a need for universal provision of engaging infant nutrition practices, but particularly to families living in areas of deprivation. However, as shown in this systematic review, the desire to include families is not enough, as many families in need of these interventions come from deprived areas and may have cultural barriers or accessibility issues to overcome in order to be involved. Three studies provided monetary incentives or a stipend to parents which enabled them to participate, and one study utilised participatory research where parents were involved in designing the study. There is a need to address the challenge of vulnerable parents receiving the necessary interventions to prevent childhood obesity. Educational workshops for parents and young children focusing on diet and activity should become standard practice. This would reduce the number of children starting school overweight or obese and encountering the incumbent problems of being an obese child.

## Supplemental Material

CHCA_10.1177_1367493520917863 – Interventions that prevent or reduce obesity in children from birth to five years of age: A systematic review Click here for additional data file. Supplemental Material, CHCA_10.1177_1367493520917863 for Interventions that prevent or reduce obesity in children from birth to five years of age: A systematic review by Katia Narzisi and Joan Simons in Journal of Child Health Care
